# Boron carbide, B_13-*x*_C_2-*y*_ (*x* = 0.12, *y* = 0.01)

**DOI:** 10.1107/S1600536812033132

**Published:** 2012-07-28

**Authors:** Oksana Sologub, Yuichi Michiue, Takao Mori

**Affiliations:** a National Institute for Materials Science, Namiki 1-1, 305-0044 Tsukuba, Japan

## Abstract

Boron carbide phases exist over a widely varying compos­itional range B_12+*x*_C_3-*x*_ (0.06 < *x* < 1.7). One idealized structure corresponds to the B_13_C_2_ composition (space group *R*-3*m*) and contains one icosa­hedral B_12_ unit and one linear C—B—C chain. The B_12_ units are composed of crystallographically distinct B atoms B_P_ (polar, B1) and B_Eq_ (equatorial, B2). Boron icosa­hedra are inter­connected by C atoms *via* their B_Eq_ atoms, forming layers parallel to (001), while the B_12_ units of the adjacent layers are linked through inter­icosa­hedral B_P_—B_P_ bonds. The unique B atom (B_C_) connects the two C atoms of adjacent layers, forming a C—B—C chain along [001]. Depending on the carbon concentration, the carbon and B_P_ sites exhibit mixed B/C occupancies to varying degrees; besides, the B_C_ site shows partial occupancy. The decrease in carbon content was reported to be realized *via* an increasing number of chainless unit cells. On the basis of X-ray single-crystal refinement, we have concluded that the unit cell of the given boron-rich crystal contains following structural units: [B_12_] and [B_11_C] icosa­hedra (about 96 and 4%, respectively) and C—B—C chains (87%). Besides, there is a fraction of unit cells (13%) with the B atom located against the triangular face of a neighboring icosa­hedron formed by B_Eq_ (B2) thus rendering the formula B_0.87_(B_0.98_C_0.02_)_12_(B_0.13_C_0.87_)_2_ for the current boron carbide crystal.

## Related literature

For X-ray/neutron diffraction studies on boron carbide, see: Yakel (1975 ▶); Will *et al.* (1979 ▶); Larson (1986 ▶); Kwei & Morosin (1996 ▶). For the boron carbide homogeneity field, see: Bouchacourt & Thevenot (1985 ▶); Gosset & Colin (1991 ▶). For electronic structure and bonding properties, see: Domnich *et al.* (2011 ▶); Balakrishnarajan *et al.* (2007 ▶). For electronic properties and charge transport, see: Werheit (2009 ▶).

## Experimental

### 

#### Crystal data

C_1.99_B_12.88_
*M* *_r_* = 163.14
Trigonal, 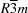

*a* = 5.6530 (8) Å
*c* = 12.156 (4) Å
*V* = 336.42 (17) Å^3^
*Z* = 3
Mo *K*α radiation
μ = 0.10 mm^−1^
*T* = 293 K
0.45 × 0.30 × 0.21 mm

#### Data collection

Rigaku AFC 7R diffractometer
1489 measured reflections
284 independent reflections
184 reflections with *I* > 2σ(*I*)
*R*_int_ = 0.151
3 standard reflections every 150 reflections intensity decay: none

#### Refinement

*R*[*F* ^2^ > 2σ(*F* ^2^)] = 0.046
*wR*(*F* ^2^) = 0.109
*S* = 1.03
284 reflections
22 parameters
1 restraint
Δρ_max_ = 0.55 e Å^−3^
Δρ_min_ = −0.37 e Å^−3^

### 

Data collection: *Rigaku/AFC Diffractometer Control Software* (Rigaku, 1998 ▶); cell refinement: *Rigaku/AFC Diffractometer Control* Software; data reduction: *TEXSAN* (Molecular Structure Corporation, 1998 ▶); program(s) used to solve structure: *SHELXS97* (Sheldrick, 2008 ▶); program(s) used to refine structure: *SHELXL97* (Sheldrick, 2008 ▶); molecular graphics: *ATOMS* (Dowty, 1999 ▶); software used to prepare material for publication: *SHELXL97* and *WinGX* (Farrugia, 1999 ▶).

## Supplementary Material

Crystal structure: contains datablock(s) global, I. DOI: 10.1107/S1600536812033132/ru2039sup1.cif

Structure factors: contains datablock(s) I. DOI: 10.1107/S1600536812033132/ru2039Isup2.hkl

Additional supplementary materials:  crystallographic information; 3D view; checkCIF report

## Figures and Tables

**Fig. 1.**
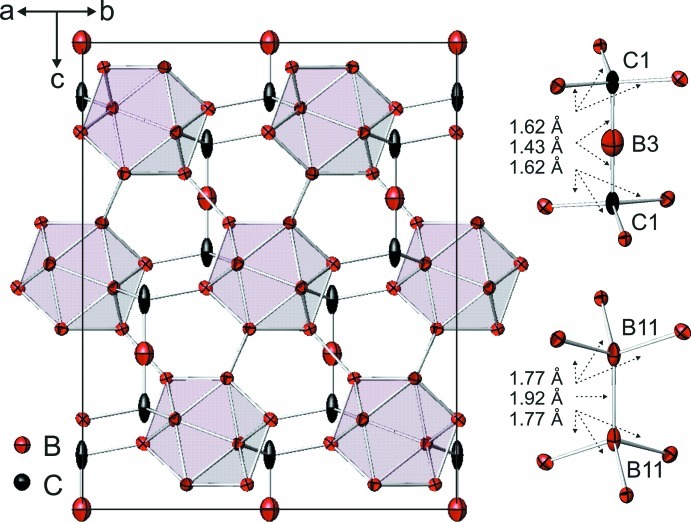
The idealized structure of boron carbide and the distances for B and C atoms of the C—B—C and B—B chains as obtained from structure refinement. Thermal ellipsoids depict the 80% probability level.

**Fig. 2.**
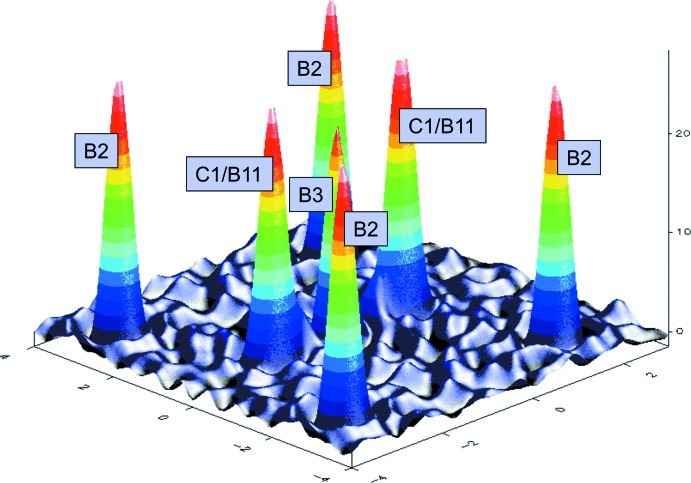
Observed Fourier maps projected onto xy plane at z=0; -1.5 e Å-3 < Δρ < 28.4 e Å-3.

## supplementary crystallographic information

### Comment

The positioning the C1 and B11 atoms on two adjacent 6c (0, 0, *z*) sites is in good agreement with observations reported from powder neutron diffraction studies of boron-rich boron carbides by Kwei and Morosin (1996). No atom in the 36i site claimed by Yakel (1975) has been found.

### Experimental

Boron carbide single-crystal has been obtained as a co-product of the yttrium boron carbide phase synthesized *via* solid state reaction of yttrium tetraboride, amorphous boron and carbon. The reaction process was performed from compacted powders in the BN crucible inserted into a graphite susceptor using the RF furnace under a flow of Ar at a temperature of about 1973 K and holding time 8 h; afterwards the setup was cooled down in 1 h to room temperature. The sample contained crystals of the title compound, in the presence of YB_28.5_C_4_ and binary yttrium borides, as revealed by powder X-ray diffraction analysis.

### Refinement

The crystal structure refinement was performed starting from the atomic coordinates reported for α-rh B. The chain atoms were located from the difference Fourier synthesis. The refinement on boron icosahedral polar site-occupancy factors led to reliability factors R1=0.0517 and wR2=0.1519 revealing the remaining electron density of 1.42 e Å^-3^ in 6c Wyckoff position (0, 0, *z*; *z*=0.07) at close distance from chain atom C1. Further refinement on occupancy parameters of the B3 (B_C_) chain atom and refining the C1/B11 in split 6c atom site reduced the highest Fourier difference peak to 0.55 e Å^-3^ at (0, 0, 0.1663) located 0.6 Å away from C1 and decreased the reliability factors to R1=0.0455 and wR2=0.1087. The ADPs of B1 and B2 have comparable values, while the thermal ellipsoids of C1/B11 chain atoms slightly extend parallel to the chain direction. The B3 in the center of a chain shows rather large ADP values. Data collection and cell refinement:

### Crystal data

**Table:**

C_1.99_B_12.88_	*D*_x_ = 2.416 Mg m^−^^3^
*M**_r_* = 163.14	Mo *K*α radiation, λ = 0.71073 Å
Trigonal, *R*3*m*	Cell parameters from 20 reflections
Hall symbol: -R 3 2"	θ = 8–50°
*a* = 5.6530 (8) Å	µ = 0.10 mm^−^^1^
*c* = 12.156 (4) Å	*T* = 293 K
*V* = 336.42 (17) Å^3^	Prism, black
*Z* = 3	0.45 × 0.3 × 0.21 mm
*F*(000) = 229	

### Data collection

**Table:**

Rigaku AFC 7R diffractometer	θ_max_ = 39.8°, θ_min_ = 4.5°
ω–2θ scans	*h* = −10→8
1489 measured reflections	*k* = 0→10
284 independent reflections	*l* = −21→21
184 reflections with *I* > 2σ(*I*)	3 standard reflections every 150 reflections
*R*_int_ = 0.151	intensity decay: none

### Refinement

**Table:**

Refinement on *F*^2^	22 parameters
Least-squares matrix: full	1 restraint
*R*[*F*^2^ > 2σ(*F*^2^)] = 0.046	*w* = 1/[σ^2^(*F*_o_^2^) + (0.0481*P*)^2^ + 0.3407*P*] where *P* = (*F*_o_^2^ + 2*F*_c_^2^)/3
*wR*(*F*^2^) = 0.109	(Δ/σ)_max_ = 0.002
*S* = 1.03	Δρ_max_ = 0.55 e Å^−^^3^
284 reflections	Δρ_min_ = −0.37 e Å^−^^3^

### Special details

**Table:**

Geometry. All e.s.d.'s (except the e.s.d. in the dihedral angle between two l.s. planes) are estimated using the full covariance matrix. The cell e.s.d.'s are taken into account individually in the estimation of e.s.d.'s in distances, angles and torsion angles; correlations between e.s.d.'s in cell parameters are only used when they are defined by crystal symmetry. An approximate (isotropic) treatment of cell e.s.d.'s is used for estimating e.s.d.'s involving l.s. planes.
Refinement. Refinement of *F*^2^ against ALL reflections. The weighted *R*-factor *wR* and goodness of fit *S* are based on *F*^2^, conventional *R*-factors *R* are based on *F*, with *F* set to zero for negative *F*^2^. The threshold expression of *F*^2^ > σ(*F*^2^) is used only for calculating *R*-factors(gt) *etc*. and is not relevant to the choice of reflections for refinement. *R*-factors based on *F*^2^ are statistically about twice as large as those based on *F*, and *R*- factors based on ALL data will be even larger.

### Fractional atomic coordinates and isotropic or equivalent isotropic displacement parameters (Å^2^)

**Table:**

	*x*	*y*	*z*	*U*_iso_*/*U*_eq_	Occ. (<1)
B1	0.44092 (16)	0.55908 (16)	0.05298 (12)	0.0053 (3)	0.958 (4)
C11	0.44092 (16)	0.55908 (16)	0.05298 (12)	0.0053 (3)	0.042 (4)
B2	0.50336 (16)	0.49664 (16)	0.19232 (11)	0.0054 (3)	
B3	0	0	0	0.0118 (8)	0.87
C1	0	0	0.1177 (5)	0.0071 (8)	0.87 (2)
B11	0	0	0.079 (4)	0.0071 (8)	0.13 (2)

### Atomic displacement parameters (Å^2^)

**Table:**

	*U*^11^	*U*^22^	*U*^33^	*U*^12^	*U*^13^	*U*^23^
B1	0.0056 (4)	0.0056 (4)	0.0047 (5)	0.0028 (5)	−0.0001 (2)	0.0001 (2)
C11	0.0056 (4)	0.0056 (4)	0.0047 (5)	0.0028 (5)	−0.0001 (2)	0.0001 (2)
B2	0.0046 (4)	0.0046 (4)	0.0059 (5)	0.0015 (4)	−0.0003 (2)	0.0003 (2)
B3	0.0099 (11)	0.0099 (11)	0.016 (2)	0.0049 (6)	0	0
C1	0.0041 (6)	0.0041 (6)	0.013 (2)	0.0021 (3)	0	0
B11	0.0041 (6)	0.0041 (6)	0.013 (2)	0.0021 (3)	0	0

### Geometric parameters (Å, º)

**Table:**

B1—C11^i^	1.731 (3)	B2—B1^ii^	1.8091 (15)
B1—B1^i^	1.731 (3)	B2—C11^iii^	1.8091 (15)
B1—B2	1.801 (2)	B2—B1^iii^	1.8091 (15)
B1—B2^ii^	1.8091 (15)	B3—B11	0.96 (5)
B1—B2^iii^	1.8091 (15)	B3—B11^vii^	0.96 (5)
B1—B1^iv^	1.825 (3)	B3—C1^vii^	1.430 (6)
B1—C11^iv^	1.825 (3)	B3—C1	1.430 (6)
B1—C11^v^	1.825 (3)	C1—B2^vi^	1.6239 (19)
B1—B1^v^	1.825 (3)	C1—B2^iii^	1.6239 (19)
B2—C1^vi^	1.6239 (19)	C1—B2^viii^	1.6239 (19)
B2—B11^vi^	1.77 (2)	B11—B2^vi^	1.77 (2)
B2—B2^iii^	1.7778 (17)	B11—B2^iii^	1.77 (2)
B2—B2^ii^	1.7778 (17)	B11—B2^viii^	1.77 (2)
B2—C11^ii^	1.8091 (15)	B11—B11^vii^	1.92 (9)
			
C11^i^—B1—B1^i^	0.00 (8)	B11^vi^—B2—C11^ii^	108.5 (11)
C11^i^—B1—B2	118.22 (14)	B2^iii^—B2—C11^ii^	108.78 (7)
B1^i^—B1—B2	118.22 (14)	B2^ii^—B2—C11^ii^	60.26 (7)
C11^i^—B1—B2^ii^	121.45 (8)	B1—B2—C11^ii^	110.05 (9)
B1^i^—B1—B2^ii^	121.45 (8)	C1^vi^—B2—B1^ii^	121.07 (19)
B2—B1—B2^ii^	59.01 (5)	B11^vi^—B2—B1^ii^	108.5 (11)
C11^i^—B1—B2^iii^	121.45 (8)	B2^iii^—B2—B1^ii^	108.78 (7)
B1^i^—B1—B2^iii^	121.45 (8)	B2^ii^—B2—B1^ii^	60.26 (7)
B2—B1—B2^iii^	59.01 (5)	B1—B2—B1^ii^	110.05 (9)
B2^ii^—B1—B2^iii^	105.68 (11)	C11^ii^—B2—B1^ii^	0.00 (11)
C11^i^—B1—B1^iv^	125.36 (8)	C1^vi^—B2—C11^iii^	121.07 (19)
B1^i^—B1—B1^iv^	125.36 (8)	B11^vi^—B2—C11^iii^	108.5 (11)
B2—B1—B1^iv^	107.10 (6)	B2^iii^—B2—C11^iii^	60.26 (7)
B2^ii^—B1—B1^iv^	107.02 (6)	B2^ii^—B2—C11^iii^	108.78 (7)
B2^iii^—B1—B1^iv^	59.72 (5)	B1—B2—C11^iii^	110.05 (9)
C11^i^—B1—C11^iv^	125.36 (8)	C11^ii^—B2—C11^iii^	60.56 (11)
B1^i^—B1—C11^iv^	125.36 (8)	B1^ii^—B2—C11^iii^	60.56 (11)
B2—B1—C11^iv^	107.10 (6)	C1^vi^—B2—B1^iii^	121.07 (19)
B2^ii^—B1—C11^iv^	107.02 (6)	B11^vi^—B2—B1^iii^	108.5 (11)
B2^iii^—B1—C11^iv^	59.72 (5)	B2^iii^—B2—B1^iii^	60.26 (7)
B1^iv^—B1—C11^iv^	0.00 (9)	B2^ii^—B2—B1^iii^	108.78 (7)
C11^i^—B1—C11^v^	125.36 (8)	B1—B2—B1^iii^	110.05 (9)
B1^i^—B1—C11^v^	125.36 (8)	C11^ii^—B2—B1^iii^	60.56 (11)
B2—B1—C11^v^	107.10 (6)	B1^ii^—B2—B1^iii^	60.56 (11)
B2^ii^—B1—C11^v^	59.72 (5)	C11^iii^—B2—B1^iii^	0.00 (4)
B2^iii^—B1—C11^v^	107.02 (6)	B11—B3—B11^vii^	180.0000 (10)
B1^iv^—B1—C11^v^	60	B11—B3—C1^vii^	180.0000 (10)
C11^iv^—B1—C11^v^	60	B11^vii^—B3—C1^vii^	0.0000 (10)
C11^i^—B1—B1^v^	125.36 (8)	B11—B3—C1	0.0000 (10)
B1^i^—B1—B1^v^	125.36 (8)	B11^vii^—B3—C1	180.0000 (10)
B2—B1—B1^v^	107.10 (6)	C1^vii^—B3—C1	180
B2^ii^—B1—B1^v^	59.72 (5)	B3—C1—B2^vi^	100.1 (2)
B2^iii^—B1—B1^v^	107.02 (6)	B3—C1—B2^iii^	100.1 (2)
B1^iv^—B1—B1^v^	60	B2^vi^—C1—B2^iii^	117.01 (13)
C11^iv^—B1—B1^v^	60	B3—C1—B2^viii^	100.1 (2)
C11^v^—B1—B1^v^	0.00 (10)	B2^vi^—C1—B2^viii^	117.01 (13)
C1^vi^—B2—B11^vi^	15.1 (12)	B2^iii^—C1—B2^viii^	117.01 (13)
C1^vi^—B2—B2^iii^	121.49 (7)	B3—B11—B2^vi^	115.2 (13)
B11^vi^—B2—B2^iii^	125.0 (2)	B3—B11—B2^iii^	115.2 (13)
C1^vi^—B2—B2^ii^	121.49 (7)	B2^vi^—B11—B2^iii^	103.2 (16)
B11^vi^—B2—B2^ii^	125.0 (2)	B3—B11—B2^viii^	115.2 (13)
B2^iii^—B2—B2^ii^	108.39 (8)	B2^vi^—B11—B2^viii^	103.2 (16)
C1^vi^—B2—B1	119.9 (2)	B2^iii^—B11—B2^viii^	103.2 (16)
B11^vi^—B2—B1	135.0 (14)	B3—B11—B11^vii^	0.0000 (10)
B2^iii^—B2—B1	60.73 (7)	B2^vi^—B11—B11^vii^	115.2 (13)
B2^ii^—B2—B1	60.73 (7)	B2^iii^—B11—B11^vii^	115.2 (13)
C1^vi^—B2—C11^ii^	121.07 (19)	B2^viii^—B11—B11^vii^	115.2 (13)

Symmetry codes: (i) −*x*+1, −*y*+1, −*z*; (ii) *x*−*y*+2/3, *x*+1/3, −*z*+1/3; (iii) *y*−1/3, −*x*+*y*+1/3, −*z*+1/3; (iv) −*x*+*y*, −*x*+1, *z*; (v) −*y*+1, *x*−*y*+1, *z*; (vi) −*x*+2/3, −*y*+1/3, −*z*+1/3; (vii) −*x*, −*y*, −*z*; (viii) *x*−*y*−1/3, *x*−2/3, −*z*+1/3.
